# Use of dermatologic ultrasound for diagnosis and surgical planning of a cutaneous arteriovenous malformation of the supratrochlear artery: A case report

**DOI:** 10.1016/j.jdcr.2025.02.019

**Published:** 2025-03-27

**Authors:** Aaron Cheng, Pavela Bambekova, Brooke A. Burgess, Nilam J. Soni, Joshua L. Owen

**Affiliations:** a Long School of Medicine, University of Texas Health San Antonio, San Antonio, Texas; b Division of Dermatology, University of Texas Health San Antonio, San Antonio, Texas; c Dermatology Service, South Texas Veterans Health Care System, San Antonio, Texas; d Medicine Service, South Texas Veterans Health Care System, San Antonio, Texas; e Department of Medicine, University of Texas Health San Antonio, San Antonio, Texas

**Keywords:** cutaneous arteriovenous malformation, dermatologic surgery, imaging in dermatology, point-of-care ultrasound, ultrasound in dermatology

## Introduction

Arteriovenous malformations (AVMs) are fast-flow vascular anomalies in which arteries directly shunt to veins.^1^ Extracranial AVMs are uncommon, and when present, typically appear on the head and neck.^2^ Clinically, cutaneous AVMs present as a violaceous patch, plaque, or nodule that might exhibit warmth, pulsatility, or audible bruit upon auscultation. AVMs progressively enlarge, are often painful, and can ulcerate, bleed, and become disfiguring. Extensive cutaneous AVMs can be complicated by high-output heart failure.^2^

Radiographic imaging modalities such as magnetic resonance imaging and computed tomography angiography can provide detailed information regarding lesion size, adjacent anatomic involvement, and vessel of origin.^3^ Point-of-care ultrasound, specifically dermatologic ultrasound (US), is a high-frequency imaging modality that can be used with or without Doppler to evaluate cutaneous AVM characteristics and aid in clinical decision-making.

We present a case of a 65-year-old male with an enlarging, intermittently bleeding nodule of the left medial eyebrow. Doppler US-assisted in diagnosing an AVM and identifying the supratrochlear artery as the vessel of origin, which informed subsequent surgical management.

## Case report

**Fig 1:**
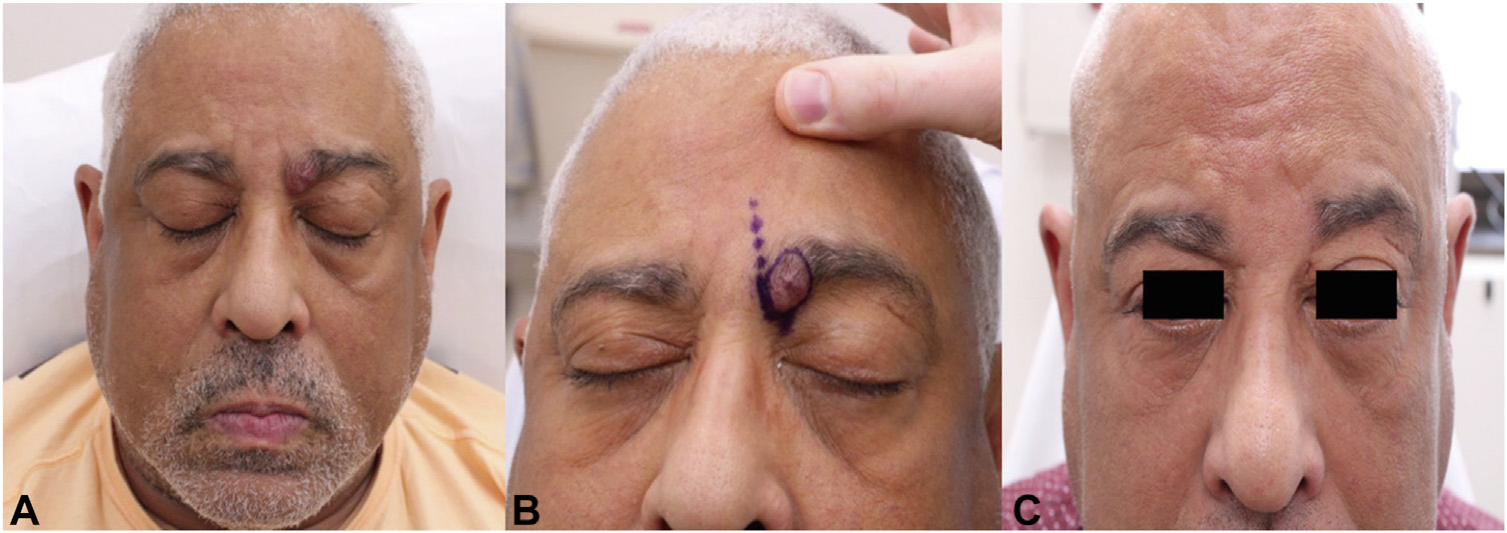
**A,** Preoperative clinical photograph exhibiting a violaceous, firm nodule on the left medial eyebrow without ulceration or active bleeding. **B,** Clinical photograph of presurgical gentian *violet* markings of the lesion’s borders (*solid circle*). *Dot* inferior to *circle* represents site of supratrochlear artery. *Dotted line* superior to *circle* marks glabellar rhytid as the site of desired placement of incision line for linear closure. **C,** Clinical photograph of surgical site 1 year postoperatively with no evidence of lesion recurrence.

A 65-year-old male presented with a slowly enlarging nodule of the left medial eyebrow that had been present since childhood. The patient reported episodes of spontaneous, significant bleeding that obstructed vision in his left eye. Physical exam revealed a violaceous, smooth, nontender nodule on the left medial eyebrow (Fig 1, *A*) with no crusting or active bleeding.

**Fig 2:**
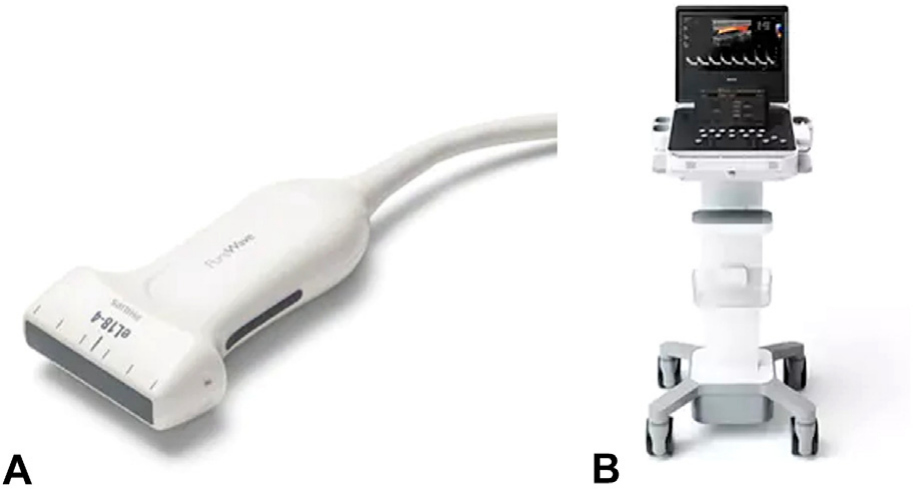
**A,** Phillips eL 18-4 PureWave transducer. **B,** Phillips Compact Ultrasound 5000 series.

**Fig 3:**
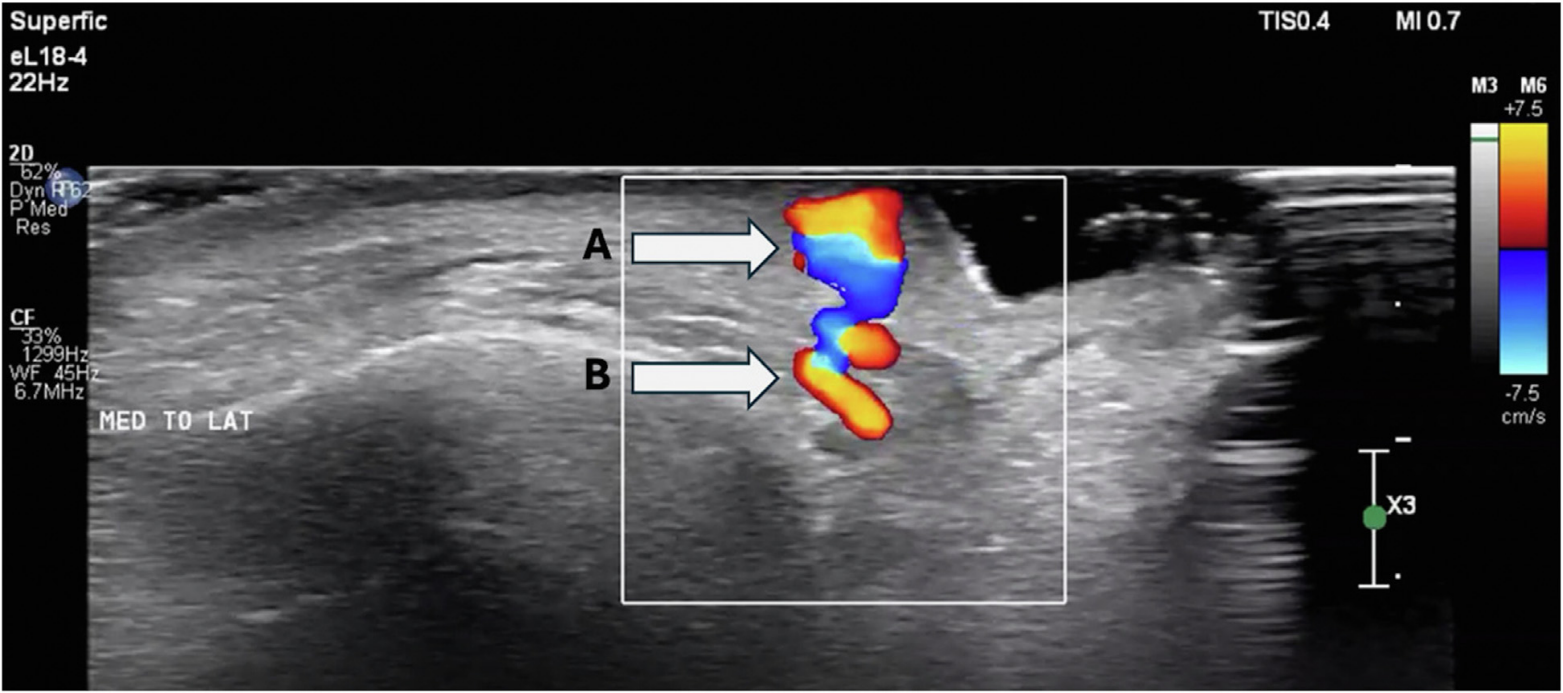
Doppler ultrasound image of the left medial eyebrow AVM using a high-frequency transducer (Phillips 5500 eL 18-4 transducer) revealing prominent bidirectional vascular flow of the (**A**) AVM and (**B**) supratrochlear artery. *AVM*, Arteriovenous malformation.

Maxillofacial computed tomography with and without intravenous contrast revealed nonspecific focal soft tissue thickening and enhancement concentrated at the left medial eyebrow with no overt underlying mass or bony erosion. US was performed utilizing a high-frequency transducer (Phillips 5500 eL 18-4 transducer; Fig 2, *A* and *B*) in clinic, revealing a well-circumscribed nodule superficial to the left orbicularis oculi muscle with high velocity, bidirectional blood flow on Doppler (Fig 3 and Supplementary Video, available via Mendeley at https://data.mendeley.com/datasets/zzy9h5njyz/1). The supratrochlear artery was visualized and appeared to be contiguous with the vascular lesion. The surrounding tissue appeared normal. Given these findings and clinical history, a diagnosis of supratrochlear AVM was made.

**Fig 4:**
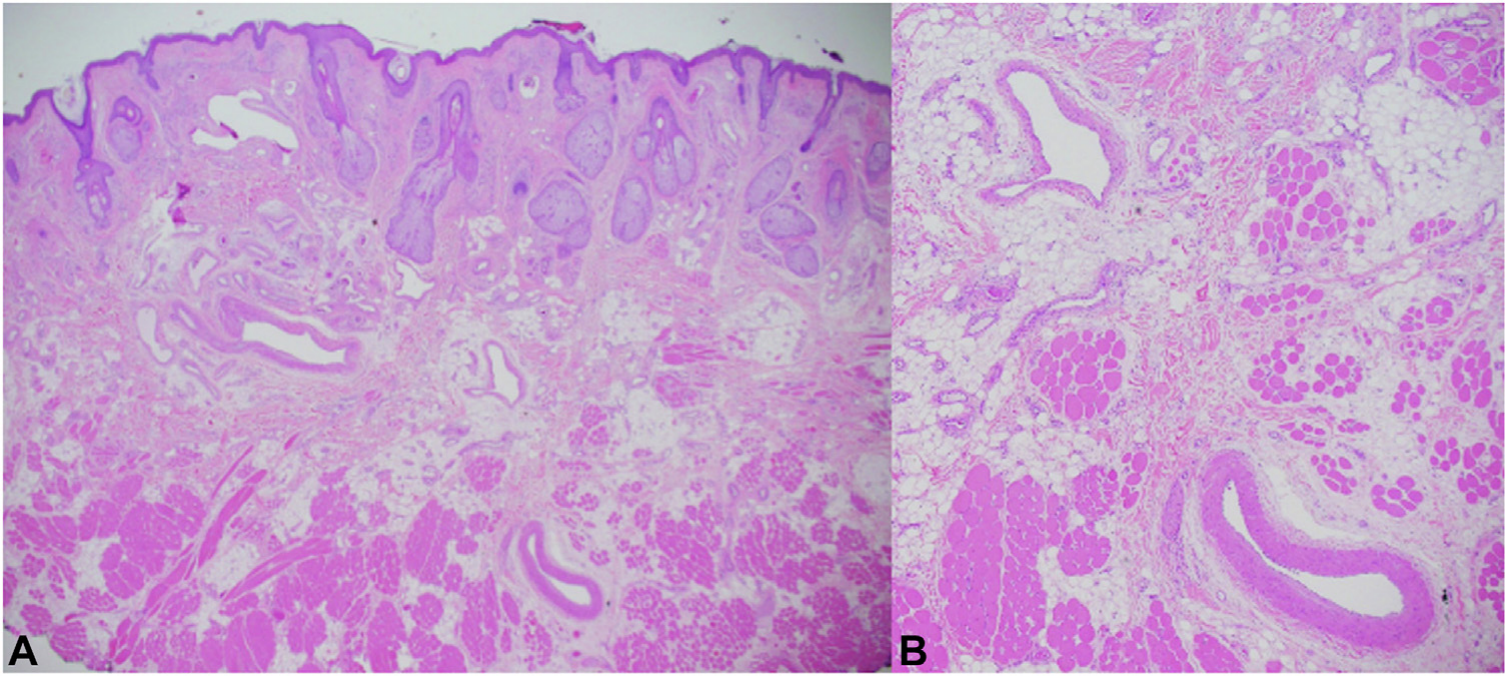
Histopathology of the supratrochlear AVM depicting haphazard proliferation of elastic arteries and thick-walled veins among adipose tissue and fibrous stroma (hematoxylin and eosin stain; **A**: 2×, **B**: 4×). *AVM*, Arteriovenous malformation.

Given the US findings, the lesion was not biopsied and instead underwent outpatient excision under local anesthesia based upon its well-circumscribed and superficial nature. First, the supratrochlear artery was identified inferior to the AVM (Fig 1, *B*) and ligated using 4-0 polyglactin 910. Second, the lesion was excised with narrow clinical margins and minimal bleeding. The resultant defect was repaired in a layered linear fashion within a glabellar rhytid. The specimen was submitted for histopathologic evaluation which was significant for a proliferation of large caliber elastic arteries and thick-walled veins with adipose tissue and fibrous stroma (Fig 4, *A* and *B*), confirming the diagnosis of AVM. One year postoperatively, the surgical site was well-healed with no evidence of AVM persistence or recurrence (Fig 1, *C*).

## Discussion

Cutaneous AVMs are typically solitary, nonsyndromic, and arise due to somatic *MAP2K1* mutations within endothelial cells.^4^ AVMs can exhibit local and systemic complications including local tissue destruction, ulceration/hemorrhage, and less commonly high-output heart failure.^3^

The clinical presentation of AVMs is variable, as they mimic other benign and malignant lesions, and diagnosis solely on clinical appearance is a challenge. A combination of clinical and radiographic data is often necessary for diagnosis and details regarding the characteristics of the AVM (eg, size, depth, and vessels involved) to help determine the best course of treatment. Imaging modalities such as magnetic resonance imaging or computed tomography angiography are often utilized but add costs and inconvenience to the patient, as well as exposure to radiation and intravenous contrast.

An alternative is the dermatologic US, a point-of-care imaging modality that uses high-frequency ultrasound waves (greater than 15 MHz) for detailed visualization of cutaneous and subcutaneous tissues.^5^^,^^6^ It is particularly useful for cutaneous vascular lesions, as Doppler ultrasound provides a dynamic assessment of the degree of vascularity and blood flow velocity.^7^ Dermatologic US is inexpensive and efficient as a part of the physical exam, can obviate the need for biopsy or other costly imaging, and facilitates shared decision-making with the patient. Our group previously reported that US is largely underutilized by dermatologists based on a national survey of Veterans Affairs medical centers.^8^

For this case, US proved to be the most appropriate imaging modality to guide clinical decision-making, when compared to computed tomography. Point-of-care US confirmed AVM diagnosis by morphology and Doppler flow, demonstrated that the lesion was superficial and well-circumscribed, and identified a connection to the supratrochlear artery. This information was reviewed with the patient in real-time, and he decided to have the AVM excised in our outpatient clinic.

Definitive treatment of AVMs can involve sclerosants, embolization, surgical excision, or a combination of embolization and surgical excision.^3^ The most appropriate form of management is largely dependent upon an accurate assessment of AVM size, local tissue destruction, and identification of the vessel of origin. If large, poorly demarcated, or fed by a vessel that cannot be easily managed intraoperatively, preoperative embolization of the AVM by interventional radiology might be necessary and outpatient excision might not be feasible. As exemplified by this case, the information provided by US resulted in the preplanned intraoperative ligation of the supratrochlear artery prior to AVM extirpation. Had the lesion been larger, invading into underlying structures, or poorly defined on US, preoperative embolization and/or general anesthesia for excision and repair would likely have been necessary.

In conclusion, early diagnosis and comprehensive evaluation of AVMs is crucial to select the most appropriate treatment modality. US played an invaluable role in this case in terms of diagnosis, shared decision-making, and surgical planning. US, despite being time- and cost-effective, remains an underutilized diagnostic modality in dermatology; if adopted more broadly, US has the potential to significantly enhance patient care.

## Conflicts of interest

None disclosed.
